# Screening for social determinants of health in clinical care: moving from the margins to the mainstream

**DOI:** 10.1186/s40985-018-0094-7

**Published:** 2018-06-22

**Authors:** Anne Andermann

**Affiliations:** 1 St Mary’s Research Centre, McGill University, Montréal, Canada; 2 Department of Family Medicine and Department of Epidemiology, Biostatistics and Occupational Health, McGill University, Montréal, Canada

**Keywords:** Screening, Social determinants, Marginalized patients, Intersectoral action, Community oriented primary care, Secondary prevention, Review

## Abstract

**Background:**

Screening for the social determinants of health in clinical practice is still widely debated.

**Methods:**

A scoping review was used to (1) explore the various screening tools that are available to identify social risk, (2) examine the impact that screening for social determinants has on health and social outcomes, and (3) identify factors that promote the uptake of screening in routine clinical care.

**Results:**

Over the last two decades, a growing number of screening tools have been developed to help frontline health workers ask about the social determinants of health in clinical care. In addition to clinical practice guidelines that recommend screening for specific areas of social risk (e.g., violence in pregnancy), there is also a growing body of evidence exploring the use of screening or case finding for identifying multiple domains of social risk (e.g., poverty, food insecurity, violence, unemployment, and housing problems).

**Conclusion:**

There is increasing traction within the medical field for improving social history taking and integrating more formal screening for social determinants of health within clinical practice. There is also a growing number of high-quality evidence-based reviews that identify interventions that are effective in promoting health equity at the individual patient level, and at broader community and structural levels.

## Background

Determining whether the social determinants influence health is no longer a topic of debate, at least not within the health field. Landmark documents such as the 2008 *Closing the Gap* report by the World Health Organization Commission on Social Determinants of Health [1] and books such as *The Spirit Level* by Wilkinson and Pickett [2] have provided substantial evidence to adequately demonstrate that decreasing income, education, social status, and social support is correlated with increased morbidity and premature mortality (also known as the social gradient). Despite the widespread acceptance of the role of social determinants in determining health, whether or not to screen for social determinants of health in clinical care remains a topic of considerable contention.

The two main groups who are less convinced about the value of screening for social determinants of health within clinical care adopt this stance for very different reasons. On the one hand, it has been shown that while many health workers can appreciate the connection between social factors and poor health, common themes explaining their reticence to ask about and address social determinants include being overworked, not knowing how to ask about social determinants or what to do about it once they find out, questioning whether addressing social determinants is part of their role, lacking role models and support in helping patients address the social determinants, being fearful of opening a “Pandora’s box” by embarking on this path, and feeling helpless or powerless in the face of such daunting social challenges [3]. A survey conducted by the Robert Wood Johnson Foundation found that four out of five physicians do not feel confident in their capacity to meet their patients’ social needs, and they believe this impedes their ability to provide quality care [4].

On the other hand, there are also champions in the field of social determinants who question whether screening is the most appropriate level of intervention. These experts in the field rightly point out that making an impact on social determinants requires broad intersectoral action and whole of government approaches [5]. The factors influencing people’s daily living conditions are generally political and structural [6]. These experts therefore question what value, if any, talking to patients about these issues could possibly do to change the larger political and structural forces at play within a society. They consider that action on the social determinants must occur beyond the health sector, but perhaps do not sufficiently appreciate the potential catalyzing role of frontline health workers in advocating and partnering for broader social change, whether at the grassroots community level or at the broader societal level nationally and globally [7]. Indeed, there are many examples of the important influence of physician advocates in many spheres that affect health, from raising awareness on climate change to the 2017 Nobel Peace Prize being awarded to an initiative launched by International Physicians for the Prevention of Nuclear War.

There is growing interest among frontline health workers, particularly, but not limited to, those working in areas such as immigrant and refugee health, caring for homeless and marginally housed persons, inner city health, Indigenous health, social pediatrics, cultural psychiatry, community-oriented primary care and global health, who want to be equipped with evidence-based guidance on how to better care for and support marginalized populations as part of their day-to-day clinical practice. Indeed, with the Lancet Commission on the Education of Health Professionals for the 21st Century highlighting the need for increasing emphasis on social accountability in medical education [8], as well as expanding networks of equity-focused medical educators such as Towards Unity for Health (TUFH), there is a strong core group of health professionals wanting to be more proactive when it comes to addressing social determinants in clinical care.

The purpose of this review is therefore to examine the evidence relating to screening for the social determinants of health in clinical care, including identifying (1) what screening tools currently exist, (2) the potential impact screening can have on improving patient outcomes (i.e., effectiveness), and (3) what factors promote health worker uptake and offer of screening in clinical settings (i.e., adherence).

## Methods

**Fig. 1:**
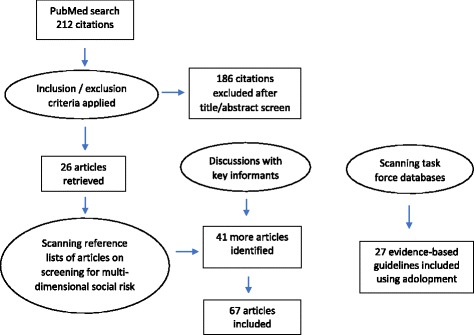
PRISMA flow diagram of articles included in the review

The scoping review followed commonly used methodology as described elsewhere [9]. A search strategy using key search terms relating to social determinants of health and screening (Table 1) were used to identify primary and secondary research studies in PubMed (MEDLINE). In total, there were 212 publications identified (Fig. 1). Titles and abstracts were scanned for relevance, and a total of 26 articles were retained. Inclusion criteria consisted of (a) English-language studies from 1970 to the present reporting on the findings of a primary or secondary research study, (b) the main focus of the study relates to screening or systematic case finding of patients with one or more social risk factors (e.g., food insecurity, exposure to violence, poverty), (c) the study involves screening or case finding that is carried out by health workers in a clinical care setting (as opposed to a population-based program), and (d) the publication reports on the types of screening tools used, the impacts of screening, and/or the factors affecting uptake and adherence to screening in clinical care. Exclusion criteria included the following: (a) the main intervention(s) under study do not involve secondary prevention (i.e., screening) or (b) the main aim of screening does not involve identifying social risk. Data extraction was carried out on the 26 retained articles, and findings were grouped according to predetermined and emerging themes using a deductive-inductive approach [10]. The main themes in the deductive framework included the following: (a) how to screen for social risk, (b) what health and social outcomes are impacted as a result of screening, and (c) what are the barriers and facilitators for frontline health workers in adopting social risk screening.

**Table 1:** Initial search strategy used for scoping review

(("social determinants of health"[mesh] OR social determinant*[ti]) OR ((violence[mesh] OR violence[ti] AND (health care[ti] OR health services[ti] OR health sector*[ti] OR healthcare[ti]))) OR ("Food Supply"[Mesh]) OR (food insecurit*[ti]) OR (poverty[mesh] OR poverty[ti]) OR (unemployment[mesh] OR unemploy*[ti]) OR (low income[ti]) OR (underemploy*[ti]) OR ("social isolation"[MeSH Terms]) OR (social exclusion[ti] OR social isolation[ti] OR socially isolated[ti] OR socially excluded[ti]) OR (support network*[ti] OR social support[ti] OR social network*[ti]) OR ("Social Environment"[Mesh]) OR ("housing"[MeSH Terms]) OR “homeless persons”[mesh] OR (homeless*[ti]) OR (hunger[ti])) AND ("mass screening"[mesh] OR screen*[ti] OR secondary prevention[mesh] OR (prevent*[ti] AND (service*[ti] OR care[ti] OR healthcare[ti])) OR social history taking[tw] OR preventive practice*[tw]) AND (practice guidelines as topic[mesh] OR practice guideline[publication type] OR guideline*[ti] OR systematic[sb] OR evidence informed[ti] OR evidence based[ti] OR recommendation*[ti] OR statement*[ti])

However, as this is an emerging field of research, discussions with key informants and key word searches in Google Scholar were used to identify the grey literature. As well, reference lists of the articles relating to multi-dimensional social risk screening, where there is a dearth of research studies, were scanned to identify other relevant articles using a snowball technique. In this way, a further 41 publications were identified, and data extraction and synthesis was also carried out as detailed above.

Finally, adolopment was used to incorporate evidence that has already been synthesized from previous systematic reviews and meta-analyses. Four evidence-based databases were scanned for high-quality reviews relating to screening for social determinants in clinical care, or related interventions to improve health equity, where the recommendations are to “recommend” or “strongly recommend” the intervention based on moderate- or high-quality evidence. The databases searched include The Canadian Task Force on Preventive Health Care, the US Preventive Services Task Force, the NICE UK database, and the Guide to Community Preventive Services. In total, 27 evidence-based reviews were identified, and these interventions are summarized in a separate table.

## Results

Much of the growing literature on screening for social determinants of health in clinical practice stems from research in the field of pediatrics, and to a lesser extent in obstetrics and family medicine (Table 2). The majority of these studies were carried out in high-income country contexts, and there is a preponderance of research on screening for violence in particular, with considerably less published in areas such as screening for food insecurity, employment, and housing stability.

**Table 2:** Articles included in the review

Publication	Topic area
Aery et al. 2017	Screening tools
Gallione et al. 2017	Screening tools
Morone et al. 2017	Screening tools
Thomas et al. 2017	Screening tools
Pai et al. 2016	Screening tools
Cohen-Silver et al. 2016	Screening tools
Andermann et al. 2015	Screening tools
Bright et al. 2015	Screening tools
Behforouz et al. 2014	Screening tools
Elbogen et al. 2014	Screening tools
Soc Adol Health and Med, 2013	Screening tools
Vogel, 2013	Screening tools
Hawkins et al. 2012	Screening tools
Bricic et al. 2011	Screening tools
Phelan, 2010	Screening tools
Roffman et al. 2008	Screening tools
Denny, 2007	Screening tools
Olive, 2007	Screening tools
Harley, 2006	Screening tools
Wilson et al. 2006	Screening tools
Savell, 2005	Screening tools
Lapp, 2000	Screening tools
Cohen et al. 1991	Screening tools
Sprague et al. 2016	Effectiveness—single domain
Strong et al. 2016	Effectiveness—single domain
Williams et al. 2016	Effectiveness—single domain
O’Doherty et al. 2015	Effectiveness—single domain
O’Doherty et al. 2014	Effectiveness—single domain
Taft et al. 2013	Effectiveness—single domain
Decker et al. 2012	Effectiveness—single domain
Taft et al. 2012	Effectiveness—single domain
Zibowski et al. 2012	Effectiveness—single domain
Feder et al. 2009	Effectiveness—single domain
Killick et al. 2009	Effectiveness—single domain
Beautrais et al. 2007	Effectiveness—single domain
Holland and Bultz, 2007	Effectiveness—single domain
Trabold, 2007	Effectiveness—single domain
Bilukha et al. 2005	Effectiveness—single domain
Mulvihill, 2005	Effectiveness—single domain
Taket, 2004	Effectiveness—single domain
Malecha, 2003	Effectiveness—single domain
Wathen et al. 2003	Effectiveness—single domain
Anderson et al. 2002	Effectiveness—single domain
Godfrey, 2001	Effectiveness—single domain
MacMillan, 2000	Effectiveness—single domain
Sullivan and Bybee, 1999	Effectiveness—single domain
Chung et al. 2016	Effectiveness—multiple domains
Naz et al. 2016	Effectiveness—multiple domains
Garg et al. 2015	Effectiveness—multiple domains
Bigrigg et al. 2005	Effectiveness—multiple domains
Anderson et al. 2002	Effectiveness—multiple domains
Grant et al. 2000	Effectiveness—multiple domains
Konijnendijk et al. 2015	Adherence
Miller et al. 2015	Adherence
Konijnendijk et al. 2014	Adherence
Bellini and Quack-Lötscher, 2013	Adherence
Knox and Aspy, 2011	Adherence
O’Campo et al. 2011	Adherence
Reijneveld et al. 2008	Adherence
Mercer et al. 2007	Adherence
Stayton and Duncan, 2005	Adherence
Presley and Robinson, 2002	Adherence
Hakim and Bye, 2001	Adherence
Rapp-Paglicci and Dulmus, 2001	Adherence
Garg et al. 2016	Whether to screen
Gottlieb et al. 2016	Whether to screen
Bayer and Johns, 2016	Whether to screen
Silverstein et al. 2002	Whether to screen

The findings of the review are summarized below according to the following main themes: (1) screening tools to identify social determinants in clinical care, (2) evidence of screening effectiveness for improving health and social outcomes, (3) adherence of health professionals to screening guidelines, and (4) whether or not to screen for social determinants of health and under what circumstances (i.e., balancing the benefits versus potential harms).

### Screening tools to identify social determinants

As early as the year 2000, the American College of Obstetricians and Gynaecologists (ACOG) had developed an educational bulletin on perinatal screening and intervention for psychosocial risk factors including barriers to care, frequent moves, safety, food insecurity, substance abuse, partner violence, stress, and unintended pregnancy [11]. The College considered that addressing psychosocial issues is an important part of improving health, that screening should be performed regularly and documented in the patient chart (Table 3), and that an effective system of referrals will increase the likelihood of successful intervention.

**Table 3:** Sample psychosocial screening tool for prenatal care

Do you have any problems that prevent you from keeping your health care appointments? How many times have you moved in the past 12 months? Do you feel unsafe where you live? Do you or any members of your household go to bed hungry? In the past two months, have you used any form of tobacco? In the past two months, have you used drugs or alcohol (beer, wine or mixed drinks)? In the past year, has anyone hit you or tried to hurt you? How do you rate your current stress level—low or high? If you could change the timing of this pregnancy, would you want it to happen earlier, later, not at all or would you not change it?	

Adapted from reference [11]

Over the last two decades, a growing number of screening tools have been developed to help frontline health workers ask about social determinants of health in clinical care, ranging from identifying food insecurity in specific populations, such as in the elderly [12] or among diabetic patients [13], to screening for violence including adverse childhood experiences [14], intimate partner violence [15, 16], and elder abuse [17]. For instance, Gallione and colleagues conducted a systematic review of screening tools for elder abuse and identified 11 such tools, some of which had been tested and validated [18]. The authors conclude that the choice of which screening tool to choose depends upon the appropriateness to the clinical setting and context.

In terms of screening for violence, which is generally more developed than other social determinants of health content domains, there are publications on screening as it relates to a variety of settings, from the emergency department [19] to the operating room [20]. As well, there are screening tools not only to identify victims of violence, but also potential perpetrators [21, 22]. However, in terms of screening for social determinants of health more broadly, there are often fewer tools that cover multiple domains in a more comprehensive way. For instance, the Poverty Tool, as the name suggests, focuses primarily on screening for financial insecurity using the simple phrase “do you have trouble making ends meet at the end of the month” [23]. This tool is currently in the process of being evaluated in primary care [24].

In the field of pediatrics, Morone from the University of Pennsylvania conducted a recent review of screening tools to identify the social determinants of health in pediatric care; however, relatively few were identified, and among these, the majority focused on a limited number of domains and used heterogeneous approaches for identifying patients at increased risk [25]. Similarly, Pai and colleagues from Ontario, Canada, also conducted a review to identify social risk screening tools for pediatric inpatients, and among 44 instruments identified, 61% focused on a single social risk theme, only 18% covered more than 5 themes, and none met the criteria for a valid content and methodologically strong social risk screening instrument for hospitalized children [26]. The authors conclude that more research is needed in this area.

While not a screening tool *per se*, Behforouz and colleagues propose that what is needed is to train health workers to take a more in depth social history on all patients that includes topics relating to individual characteristics, life circumstances, emotional health, perceptions of health care, health-related behaviors, and access to and utilization of health care (Table 4) [27]. In a similar vein, the CLEAR toolkit which is available for download free of charge in over a dozen languages provides a broad overview of key domains relating to social determinants of health that can be screened for in clinical care (i.e., employment, child care, food insecurity, housing, domestic violence, child maltreatment, discrimination, and isolation) and facilitates mapping out the related referral resources, while encouraging local adaptation of how to ask the questions as well as identifying the most appropriate interventions, based on local knowledge of the specific context [28].

**Table 4:** Proposed topics for taking a more complete social history

1. Individual characteristics • Self-defined race or ethnicity • Place of birth or nationality • Primary spoken language • English literacy • Life experiences (education, job history, military service, traumatic or life-shaping experiences) • Gender identification and sexual practices 2. Life circumstances • Marital status and children • Family structure, obligations, and stresses • Housing environment and safety • Food security • Legal and immigration issues • Employment (number of jobs, work hours, stresses/concerns about work) 3. Emotional health • Emotional state and history of mental illness (e.g., depression, anxiety, trauma, post-traumatic stress) • Causes of recent and long-term stress • Positive or negative social network: individual, family, community • Religious affiliation and spiritual beliefs 4. Perception of health care • Life goals & priorities; ranking health among other life priorities • Personal sense of health or fears regarding health care • Perceived or desired role for health care providers • Perceptions of medication and medical technology • Positive or negative health care experiences • Alternative care practices • Advance directives for cardiopulmonary resuscitation 5. Health-related behaviors • Sense of healthy or unhealthy behaviors • Facilitators of health promotion (e.g., behaviors among peers) • Triggers for harmful behaviors and motivation to change (determined through motivational interviewing) • Diet and exercise habits • Facilitators or barriers to medication adherence • Tobacco, alcohol, drug use habits • Safety precautions: seatbelts, helmets, firearms, street violence 6. Access to and utilization of health care • Health insurance status • Medication access and affordability • Health literacy and numeracy • Barriers to making appointments (e.g., child care, work allowance, affordability of copayment, transportation)	

Adapted from reference [27]

For specific population subgroups, such as adolescents, the HEADSS psychosocial screening tool has been used for several decades and examines (1) the home environment, (2) education and employment, (3) activities, (4) drugs, (5) sexuality, and (6) suicide and depression [29]. Similarly, the Family First screening tool has been used in school-based medical clinics to assess (1) maternal age; (2) education, income, and employment; (3) mental health problems and addictions; (4) parental attachment; (5) marital discord; and (6) social isolation [30]. Thus, a wide range of screening tools are already in existence, though mostly in terms of assessing a single domain of social risk, with some promising examples of multi-domain tools or approaches to social history taking.

### Evidence of screening effectiveness on improving patient outcomes

The evidence of effectiveness of screening for social risk can be divided into two categories: (1) screening for single domains of social risk versus (2) simultaneously screening for multiple domains of social risk. In terms of the former, there is a much larger literature available, as well as several clinical practice guidelines, relating to screening for specific single domains of social risk (e.g., screening for intimate partner violence). However, it is known that social risks tend to cluster. Therefore, intuitively, it makes sense to simultaneously screen for multiple domains of social risk. This is an approach that has long been used by physicians in the field of social medicine, but a relatively recent focus of research inquiry with regards to mainstream care.

#### The impact of screening for a single domain of social risk

There are many evaluations of screening for single domains of social risk and, in particular, a large literature on screening for different types of violence, particularly intimate partner violence [31], as well as suicide (self harm), child abuse, and elder abuse .

While a Cochrane Review did not find sufficient evidence to support an association between screening and reduced harm to women experiencing violence [32], this does not mean that screening is not effective, simply that there is not sufficient evidence at this time to demonstrate an effect [33]. Similarly, an analysis from the UK found that the HITS (Hurts, Insults, Threatens and Screams) scale is a sensitive screening tool able to identify victims of violence in health care settings, most women consider screening for domestic violence to be acceptable, and there is growing evidence of effectiveness for advocacy and psychosocial counselling, nonetheless, universal screening of all women presenting to clinical care in the absence of violence-related concerns or health conditions does not yet meet the criteria of the National Screening Committee of the NHS [34].

Not surprisingly, the rates of screening for violence in practice across various health care settings (i.e. prenatal care and pediatrics) are variable [35]. Many in the field advocate for developing more evidence-based approaches to assist women when they do disclose abuse and for greater emphasis on training health professionals to respond appropriately to such disclosures [36]. This is important recognizing that addressing such issues in clinical care can be complex and often raises certain ethical challenges [37] and requires a broader systems approach to ensure patient-centered care, access to appropriate referral pathways, and timely follow-up [38]. Implementation science research is also needed to improve screening uptake and ensure the translation of research findings into routine practice [39].

In terms of current national guidelines, the US Preventive Services Task Force (USPSTF) recommends that health workers screen all women of childbearing age for intimate partner violence and provide or refer women who screen positive to intervention services (Grade B) [40]. The Canadian Task Force on Preventive Health Care (CTFPHC) considers that while there is insufficient evidence to recommend for or against routine universal screening for violence against either pregnant or nonpregnant women (grade I), “clinicians should be alert to signs and symptoms of potential abuse and may wish to ask about exposure to abuse during diagnostic evaluation of these patients” [41]. There is fair evidence to refer women who have spent at least 1 night in a shelter to a structured program of advocacy services [42]. The Community Preventive Services Task Force (CPSTF) as well as the USPSTF and CTFPHC all recommend the use of early childhood home visitation programs based on strong evidence of their effectiveness in reducing child abuse and neglect among high-risk families [43, 44].

In terms of youth violence, a recent systematic review found evidence that health care-based violence intervention programs reduced recidivism as well as health care and criminal justice system costs [45]. In addition, intermediate outcomes included increased service use, positive attitude change, and decreases in violence-related behavior. Regarding suicide, promising interventions likely to be effective in reducing suicidal behaviors are medical practitioner and gatekeeper education, and restriction of access to lethal means of suicide [46].

#### The impact of simultaneous screening for multiple domains of social risk

There is a small but growing body of evidence on the impact of screening for social determinants of health more broadly in clinical care. Early research has shown that health workers who feel at ease asking about social determinants of health in clinical care are more likely to report having helped their patients in addressing these issues [47].

The Task Force on Community Preventive Services has conducted systematic reviews of early childhood development interventions and family housing interventions and concluded that these interventions do effectively address sociocultural factors that influence health [48]. The Task Force strongly recommends publicly funded, center-based, comprehensive early childhood development programs for low-income children aged 3–5 years which are effective in preventing developmental delay. The Task Force also recommends housing subsidy programs for low-income families involving rental vouchers for use in the private housing market. This has been shown to improve neighborhood safety and reduce family exposure to violence.

Since this is an emerging area of research, there are mostly primary studies available including non-RCT design [49], as well as a smaller number of RCTs but focusing on diverse types of screening, with a range of different interventions and populations involved, from using patient navigators in primary care [50], to screening and referral for social risk at routine well child visits [51]. There are also a number of clinical practice guides to promote awareness among frontline clinicians, and either encourage screening [52] or at least incorporate a social determinants approach in clinical care and be aware of opportunities when it would be appropriate and good clinical practice to do so.

### Adherence of health professionals to screening guidelines

Even once there are clear national screening guidelines, implementation remains a challenge in this area which can reduce effectiveness in practice. In the Netherlands, while most clinicians were aware of guidelines to identify and address child abuse, fewer than half routinely used the guideline in clinical care, largely because they were not in the habit of doing so [53]. Factors that prevented clinicians from using the guidelines included being unaware of the content, lacking self-efficacy or confidence in being able to apply the guidelines properly, and not having pre-established linkages with referral resources [54]. Four program components that help to increase clinician self-efficacy for screening include institutional support, clear screening protocols, initial and ongoing trainings, and facilitation of access to onsite and/or offsite referral and support services [55]. Adaptation of screening protocols to different clinical settings is also important to assist clinicians in seeing the relevance of screening [56].

Another approach to promote adherence to clinical screening guidelines is to provide incentives and to make referral services more widely available. The US Affordable Care Act includes screening and brief counseling for intimate partner violence as part of required free preventive services for women [57]. There is also evidence that more time to address complex issues, even simply adding a few minutes onto the consultation, is helpful [58], and in primary health care, there is continuity of care and ongoing opportunities to address these issues over time as well as sharing the responsibility for care with a broader clinical team as well as partners working in the community in local NGOs and referral support centers.

### Whether or not to screen for social determinants of health

Already a decade ago, Silverstein and colleagues from Boston University had conducted a review on screening for social determinants of health in pediatric primary care, including identifying factors such as maternal depression, domestic and intimate partner violence, school readiness and eligibility for early learning programs, food insecurity, and housing quality and affordability [59]. The authors concluded that each of these factors is closely linked to patient health outcomes and that the growing body of evidence supports social screening and intervention in primary care, while recognizing the need to continue to develop and refine available screening tools and interventions.

More recently, Garg and colleagues warned about avoiding the unintended consequences of screening for social determinants, particularly in the absence of available referral networks to address identified social needs [60]. This sparked a great deal of debate from supporters of screening who argued that even in the absence of available programs and services, screening can help to identify patients who need more support in primary care [61] and can lay the groundwork for the future development of interventions that are better adapted to patient needs [62].

## Discussion

This review has demonstrated that over the last few decades, there has been a growing literature on screening for the social determinants of health in clinical practice. There are an increasing number of screening tools for single and multiple dimensions of social risk and also for specific populations ranging from veterans [63] to the LGBT community [64]. There are also more and more primary research studies and reviews being published that examine the efficacy and effectiveness of screening. For instance, Naz and colleagues [47] found that health workers who have sensitive and caring ways to ask about social determinants were able to open the door to addressing these issues in clinical care. A cluster RCT conducted by Garg and colleagues [51] demonstrated that screening for social determinants of health during well child care visits led to greater referral to social support resources, greater odds of being employed and having child care at 12 months of follow-up, and lower odds of being in a shelter. In addition to improving social outcomes, studies have also shown improvements in health outcomes, such as Strong and colleagues [45] who found that screening for social determinants, and particularly for violence exposure, among youth presenting with injuries led to a reduction in recurrent presentations to clinical care for repeat injuries (i.e., recidivism).

Yet, amassing a body of evidence to demonstrate sufficient benefit in a complex area such as this has resulted in some divergence in national screening recommendations even around single-dimension screening such as screening for intimate partner violence. National recommendations around multi-dimension screening for social risk are not yet available since the evidence base to support such recommendations is highly under-developed at present. More research is still needed in this area to be able to demonstrate whether screening for social risk, and especially for multiple domains of social risk, which require complex and individually tailored interventions, often developed through participatory and community-informed approaches to address local contextual factors [65] and which lead to multiple relevant outcome measures, will succeed in meeting the Wilson and Jungner screening criteria [66]. There is also an ongoing debate regarding the potential unintended consequences of screening which very much depends on how this is done and how well-trained and prepared the clinical staff are and whether referral resources have been sufficiently mapped out. Thus, while adherence to screening in the area of well-defined clinical practice guidelines already demonstrates certain challenges, as one moves towards more complex areas such as screening for social determinants of health, a far more robust evidence base will be needed to generate widespread support and health care culture change in this area.

In light of the current state of the evidence on screening for the social determinants of health, it is important for frontline clinicians to be aware that even when there is insufficient evidence to recommend universal screening of the general population of asymptomatic individuals using specific screening tools and predetermined interventions, clinicians nonetheless require training to know how to ask about social determinants and how to map out referral resources and implement other models of care (e.g., patient navigators) when social risk is relevant to the clinical presentation. Reasons to do so are multiple including (1) providing whole-person care rather than focusing only on the disease, (2) reducing missed opportunities for diagnosis by having all the important information in terms of living conditions and social context, (3) reducing “revolving door medicine” and recurrent emergency room visits by understanding and addressing the underlying causes of the presenting health issues, (4) providing more cost-effective care by intervening early and preventing hospitalization, (5) increasing adherence to medication and improving health by prescribing medicines that patients can afford and are therefore more likely to take regularly as prescribed, and (6) providing more trauma-informed and structurally competent care.

For instance, unless health workers routinely ask about exposure to violence in the work up of pelvic pain, they may be missing important opportunities for intervention and may instead embark upon a potentially iatrogenic diagnostic odyssey that misses the main factors underlying the clinical presentation [67]. Similarly, unless clinicians ask their patients whether they will be able to afford the medicines being prescribed, this can lead to medication non-compliance and worsening of the health condition [68]. Just because there may be insufficient evidence to recommend universal screening does not mean that social determinants is unimportant or should be left out of clinical care. Rather, having a heightened awareness of social determinants and enquiring about social history is part of good clinical practice that can lead to better adapted diagnosis and management in a wide range of areas.

Indeed, some would even go so far as to say there is an ethical imperative to act on the social determinants of health in clinical care. According to Rapp-Paglicci and Dulmus, medical centers in the US see 1.4 million serious violent crime victims every year; however very few medical centers evaluate patients beyond physical conditions, and even fewer complete toxicology or psychosocial screens to evaluate for substance abuse and psychological conditions as a result of trauma [69]. Victims of violence are often given medical assistance and discharged without recognition of the serious after effects of trauma for both themselves and their families, as well as the high likelihood of re-victimization..

While the evidence base in this area is still emerging, there already exist numerous examples where there is very strong evidence and widespread consensus regarding interventions that do work to take action on the social determinants of health as summarized in Table 5. Health workers can therefore already get started by incorporating a social determinants lens into their clinical practice and ensuring that patients are able to access these proven interventions ranging from early child home visitation programs for reducing child maltreatment to high school completion programs and tenant-based rental assistance programs.

**Table 5:** Evidence-based recommendations to screen for and address social determinants

1. Canadian Task Force on Preventive Health Care • Not applicable. 2. US Preventive Services Task Force Intimate partner violence • Screen women of childbearing age for intimate partner violence (IPV), such as domestic violence, and provide or refer women who screen positive to intervention services (Grade B) Alcohol misuse • Screen adults aged 18 years or older for alcohol misuse and provide persons engaged in risky or hazardous drinking with brief behavioral counseling interventions to reduce alcohol misuse (Grade B) 3. NICE UK Clinical Guidelines Violence, abuse and neglect • Child maltreatment (CG89] • Child abuse and neglect [NG76] • Domestic violence and abuse [PH50] Addictions • Alcohol-use disorders [CG115] • Drug misuse in over 16s [CG15] • Drug misuse prevention [NG64] Complex social factors in pregnancy • Pregnancy and complex social factors [CG110] Social and emotional wellbeing • The early years [PH40] • In primary education [PH12] Housing • Reducing health risks from living in a cold home [NG6] 4. Guide to Community Preventive Services (The Community Guide) Violence, abuse and neglect • Early Childhood Home Visitation to prevent child maltreatment • School-Based Programs • Therapeutic Foster Care for chronically delinquent juveniles • Cognitive-Behavioral Therapy to address psychological harm from traumatic events among children & adolescents • Interventions to improve caregivers' parenting skills Addictions • Excessive alcohol consumption - Laws prohibiting sales to minors • Alcohol-Impaired Driving: Minimum legal drinking age laws • Lower BAC Laws for young or inexperienced drivers • School-based instructional programs Education Programs to Promote Health Equity • Full-day Kindergarten programs • Center-based Early Childhood Education • Out-of-School-Time Academic Programs – Math-Focused & Reading • School-Based Health Centers • High School Completion Programs Housing • Tenant-Based Rental Assistance Programs	

Adapted from the Canadian Task Force on Preventive Health Care [73], the US Preventive Services Task Force [74], the National Institute for Health and Care Excellence UK Clinical Guidelines [75], and The Community Guide [76]

It is also important to note that screening for social risk is a form of secondary prevention. However, there exists a continuum of strategies to improve the health of populations and reduce inequities [70], from diagnosis and treatment, to the three levels of prevention, to health promotion (i.e., healthy public policies and creating supportive environments for health) [71], as well as broader intersectoral and whole of government action on the social determinants of health [72]. Beyond what can be done at the doctor-patient level, health workers can be important advocates and catalysts of broader changes to create more supportive environments for health and to change social norms, systems, and structures, to prevent these issues from occurring in the first place. This includes informing local and national policy-makers of effective interventions that do exist and ensuring that these are made available for the local population.

## Conclusion

There is a growing body of evidence in support of screening for various aspects of social risk within routine clinical care, as part of a wider continuum of strategies for improving population health and reducing health inequities. At various times in a person’s life, everyone may face challenges in one, or often multiple, domains of social risk. Screening for social determinants of health can help to identify patients who may benefit from greater support in one or more areas, thus promoting whole-person care for the entire population, and particularly for those who are marginalized and underserved.

While there is not always consistency in clinical practice guidelines for single-dimension social risk screening (e.g., for identifying intimate partner violence) due to variability of interventions studied and outcomes measured, and while a great deal more research is needed in the area of multi-dimension screening, which is most relevant to the clinical context and meeting patient needs, there already exist many effective and evidence-based interventions to promote health equity, but clinicians would need to identify patients for whom referral to these interventions would be appropriate and may also need to raise awareness and convince local policy-makers to make these interventions available in the local setting. Thus, screening for social determinants of health is an emerging area of clinical practice that still requires a great deal more research and ongoing continuing medical education on how to do this in practice.

Yet, there is increasing traction within the medical field for improving social history-taking and integrating more formal screening for social determinants of health within clinical practice. There is an increasing diversity of screening tools now available, which can be adapted and tailored to the local context, practice population, and needs. There is therefore a great deal that frontline health workers can already do to begin to address social determinants in clinical practice and beyond.
